# No Association of Multiple Sclerosis with *C9orf72* Hexanucleotide Repeat Size in an Austrian Cohort

**DOI:** 10.3390/ijms241411254

**Published:** 2023-07-09

**Authors:** Theresa König, Fritz Leutmezer, Thomas Berger, Alexander Zimprich, Christiane Schmied, Elisabeth Stögmann, Tobias Zrzavy

**Affiliations:** 1 Department of Neurology, Medical University of Vienna, 1090 Vienna, Austria; theresa.koenig@meduniwien.ac.at (T.K.); fritz.leutmezer@meduniwien.ac.at (F.L.); thomas.berger@meduniwien.ac.at (T.B.); alexander.zimprich@meduniwien.ac.at (A.Z.); christiane.schmied@meduniwien.ac.at (C.S.); tobias.zrzavy@meduniwien.ac.at (T.Z.); 2 Comprehensive Center for Clinical Neurosciences and Mental Health, Medical University of Vienna, 1090 Vienna, Austria

**Keywords:** genetic variants, frontotemporal dementia (FTD), multiple sclerosis (MS), *C9orf72* repeat expansion, intermediate repeat length, disease heterogeneity

## Abstract

Multiple Sclerosis (MS) is a common immune-mediated disorder of the central nervous system that affects young adults and is characterized by demyelination and neurodegeneration. Recent studies have associated *C9orf72* intermediate repeat expansions with MS. The objective of this study was to investigate whether *C9orf72* repeat length is associated with MS or with a specific disease course in a monocentric Austrian MS cohort. Genotyping of 382 MS patients and 643 non-neurological controls for *C9orf72* repeat expansions was performed. The study did not find a difference in the distribution of repeat numbers between controls and MS cases (median repeat units = 2; *p* = 0.39). Additionally, sub-analysis did not establish a link between intermediate repeats and MS (*p* = 0.23) and none of the patients with progressive disease course carried an intermediate allele (20–30 repeat units). Exploratory analysis for different cut-offs (of ≥7, ≥17, and ≥24) did not reveal any significant differences in allele frequencies between MS and controls. However, the study did identify a progressive MS patient with a pathogenic *C9orf72* expansion and probable co-existing behavioral variant frontotemporal dementia (bvFTD) in a retrospective chart review. In conclusion, this study did not find evidence supporting an association between *C9orf72* repeat length and MS or a specific disease course in the Austrian MS cohort. However, the identification of a progressive MS patient with a pathogenic *C9orf72* expansion and probable co-existing with FTD highlights the complexity and challenges involved in recognizing distinct neurodegenerative diseases that may co-occur in MS patients.

## 1. Introduction

Multiple Sclerosis (MS) is the most common immune-mediated disorder of the central nervous system in young adults, characterized by demyelination and neurodegeneration. The conventional view of MS pathophysiology, supported by a plethora of available data, hypothesizes that auto-reactive cells are present in the immune repertoire of a susceptible host and overcome control mechanisms, e.g., autoimmune processes being elicited by environmental causes [1,2]. However, an alternative hypothesis that has been postulated is the so-called “inside out” model, with a primary neurodegenerative event followed by an immune response as a secondary consequence [3,4].

A GGGGCC hexanucleotide repeat expansion (HRE) in the first intron of the chromosome 9 open reading frame 72 (*C9orf72*) gene was discovered to be the most frequent cause of both frontotemporal dementia (FTD) and amyotrophic lateral sclerosis (ALS), as well as mixed FTD/ALS phenotype. While repeat units of hundreds or thousands are clearly disease-causing, a clear cut-off between normal and pathogenic repeat length has not been established in *C9orf72* yet. In most studies, repeat numbers of >30 or >45 repeat units are considered pathogenic. In recent years, this variant has been linked to a broad clinical spectrum and multiple neurodegenerative diseases, as currently reviewed [5].

A possible correlation of *C9orf72* repeat expansion with multiple sclerosis (MS) has been investigated previously. These studies, all performed in Italian patients, found no association of the pathogenic *C9orf72* repeats expansion with MS pathogenesis [6,7,8]. Nevertheless, the pathogenic expansion was reported to occur in four of five patients with coexisting ALS and MS, showing a more rapid disease progression compared to patients with pure *C9orf72* ALS. [6]. Although the concurrence of the two diseases is extremely rare, the authors suggested that MS might increase the risk of the *C9orf72* mutation becoming penetrant and possibly modifies relevant pathogenic pathways. This was also discussed in a case report of a patient diagnosed with ALS carrying a *C9orf72* repeat expansion and active brain demyelinating lesions, although the patient showed no MS symptoms [9].

Of note, a study examining a large cohort of Italian patients with progressive MS patients reported a significantly more frequent occurrence of alleles with intermediate repeat size between 20 and 30 repeat units [10]. The role of intermediate repeats in disease pathogenesis is to date not entirely clear, but they have been suggested to be a susceptibility risk factor for other neurodegenerative diseases and disease-modifier [11,12,13,14].

In this study, we screened a large Austrian MS cohort for the *C9orf72* repeat expansion. Besides evaluating repeat length distribution and frequency of intermediate and pathogenic repeat alleles in MS patients, we describe a unique case with co-incidental pathogenic *C9orf72* HRE in a patient with MS and probable co-existing FTD.

## 2. Results

### 2.1. C9orf72 Repeat Length Distribution in Multiple Sclerosis

**Figure 1:**
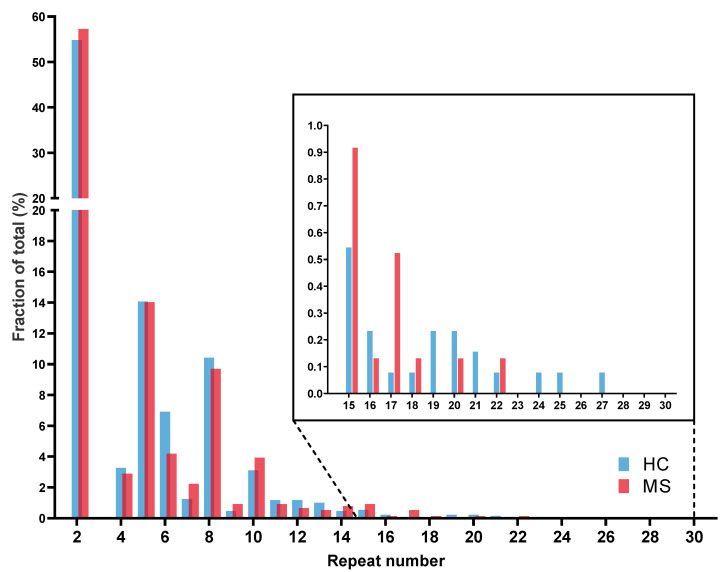
*C9orf72* repeat allele length in multiple sclerosis patients.

In the first step, the *C9orf72* repeat length distribution of the patients was compared to healthy controls. Repeat ranges of 2–27 was observed in the neurologically healthy control (HC) cohort (*n* = 643) and a range of 2–22 in the multiple sclerosis (MS) cohort (*n* = 382). Similarly, to the distribution reported previously, a characteristic trimodal pattern was observed with peaks at two, five, and eight repeat units, as shown in Figure 1. There was no difference between repeat number distribution in controls and MS cases (*p* = 0.39). Notably, we identified one patient diagnosed with secondary progressive multiple sclerosis (SPMS) carrying a repeat expansion of approximately 1300 repeat units, reported in detail below.

The histogram shows the relative frequency of repeat lengths in HC (n = 643) and MS (n = 382) cohorts. Both cohorts show a characteristic trimodal pattern with high frequency of alleles of two, five, and eight repeats. Cutout shows repeat lengths between 15 and 30 repeat units. There was no significant difference in allele frequency (Mann–Whitney U-test, *p* = 0.39).

Abbr.: HC = healthy controls; MS = multiple sclerosis.

### 2.2. C9orf72 Intermediate Allele Frequency

Considering a previous study suggesting a correlation between intermediate repeats (defined as 20–30 repeats) and primary progressive multiple sclerosis (PPMS) [10], we took a more detailed look at the occurrence of intermediate alleles and different sub-phenotypes in our cohort. Accordingly, we have classified repeat length as small (S; <20 repeats); intermediate (I; 20–30 repeats) and expanded (E; >30 repeats) alleles. Again, there was no difference between repeat lengths using these cut-offs.

Only 11 carriers of alleles ≥20 repeat units were identified, nine in the group of HC (0.7%) and two in the SPMS cohort (1.6%) without significant difference (*p* = 0.257). Mean repeat lengths and allele frequencies are shown in Table 1. Notably, the SPMS patient who was a carrier of a pathogenic *C9orf72* expansion allele was also a carrier of an intermediate repeat allele.

Additionally, other thresholds were tested, as the cut-off for the intermediate repeat length is not well established. We, therefore, assessed cut-offs of ≥7, ≥17 and ≥24, suggested in the literature based on specific risk haplotypes, studies on DNA methylation, and gene expression and association with increased risk for specific diseases [15,16,17,18,19,20]. Again, there were no significant differences in allele frequencies at those cut-offs (shown in Table 2).

Fisher’s exact test was used to compare frequencies of intermediate repeat alleles at different cut-offs using Holm–Sidak correction for multiple testing.

### 2.3. C9orf72 Hexanucleotide Repeat Expansion Carrier

Remarkably, we found one patient with a SPMS disease course carrying a pathogenic *C9orf72* repeat expansion. Southern blotting revealed an expanded allele of approximately 1300 units and an intermediate repeat of 20 units on the other allele (see Supplementary Figure S1).

The male patient was diagnosed with RRMS at the age of 28 years with no reported family history of neurodegenerative diseases. The patient was followed up regularly at the outpatient clinic of the Department of Neurology of the MUV from the age of 38 years. At the age of 45, acute worsening of symptoms occurred. The patient was admitted to the hospital after he was found completely immobile at home. At this time, a progressive deterioration of walking ability within the last two weeks was reported. The patient stated that he had recently been unable to perform hygiene adequately and that he had not continued his established Betaferon therapy for the past two weeks. The patient himself associated his symptomatology with the cold wind (“draught”). At the time of admission, clinical neurological examination revealed spastic tetraparesis and ataxia, corresponding to an EDSS (expanded disability status scale) score of 8.0. During the admission, MRI imaging of brain and spinal cord was performed, revealing multiple spinal hyperintensities as well as marked atrophy of the cervical and thoracic spinal cord. Likewise, numerous non-enhancing T2 lesions were present supratentorially on both hemispheres with generalized supratentorial and perisylvian accentuated signs of atrophy. In the neuropsychiatric examination, below-average scores were observed in Rey–Osterrieth Complex Figure Test (testing perception) and tests for divided attention. At the Wechsler Memory Scale (WMS-IV), the patient achieved below-average values in the areas of logical short- and long-term memory. Moreover, verbal fluency in terms of formal-lexical as well as semantic-categorical (Regensburger Wortflüssigkeitstest) and non-verbal fluency (HAMASCH test) was below-average. In summary, deficits in the cognitive domains: attention, short- and longer-term verbal memory, and executive functions were present. Interestingly, the patient displayed perceptual changes and mild paranoid thoughts during the inpatient stay. At the age of 46, SPMS was diagnosed. The patient committed suicide at 50 years of age, no postmortem material was available and no biomarker testing such as PET or CSF analysis specific for dementia was performed.

## 3. Discussion

In our study, we determined the *C9orf72* repeat length in an Austrian MS cohort in order to investigate the frequency of intermediate alleles ≥20 repeat units, as they were suggested to occur in higher frequency in patients diagnosed with primary progressive MS (PPMS) [10].

We did not observe a correlation of the *C9orf72* repeat length with MS or a specific disease course in our cohort. Likewise, when considering different cut-offs for intermediate repeats as previously suggested, repeat sizes between MS patients and control groups did not differ significantly in our cohort [10,21,22]. Notably, we identified one patient with a progressive disease course who was a carrier of the pathogenic *C9orf72* repeat expansion. The retrospective chart review of the medical history revealed a probable psychiatric symptomatology with the occurrence of psychotic symptoms and the committing of suicide, which is suggestive of a co-existing bvFTD pathology according to criteria of Rascovsky et al., although no post-mortem material was available [23]. The symptoms of the patient fit psychological features enriched in *C9orf72* repeat expansion carriers, which commonly include delusions and hallucinations, mood disorders, obsessive compulsive disorder and catatonia [24,25,26,27] and were reported to occur in some patients several years before characteristic ALS or FTD onset [24,27]. Atrophy patterns observed in MRI imaging of the patient were also seen in *C9orf72* patients diagnosed with FTD [28].

Pathogenic *C9orf72* repeat expansions were previously identified in patients with very rare concurrence of MS and amyotrophic lateral sclerosis (ALS), but not in patients without concomitant disease [6]. There are no reports on *C9orf72* HRE in patients with MS and coexisting FTD, although neurodegenerative dementias become more common in this diagnostic group as persons diagnosed with MS have higher life expectancy today [29,30]. However, cognitive impairment is also observed in MS patients, more pronounced at later disease stages. Cognitive impairment related to MS has shown a strong involvement of information processing speed, while other domains are less affected, including characteristic changes in neurodegenerative dementias [31]. As there are overlaps between typically affected domains, distinguishing cognitive impairment due to MS from neurodegenerative dementia co-occurring with MS based only on neuropsychiatric examination is difficult [32,33,34]. Therefore, a major limitation of this study is the lack of more detailed diagnostic work up and post-mortem tissue, as the definite diagnosis of FTD and other neurodegenerative disorders still depends on neuropathological findings in autopsy. Thus, we cannot say with certainty whether the symptoms and changes in MRI described are definitely due to the *C9orf72* repeat expansion mutation.

Furthermore, the lack of association between intermediate allele size and PPMS in our cohort could also be due to a low patient number of PPMS patients in our cohort.

In summary, our findings suggest that there is no significant contribution of intermediate *C9orf72* repeat alleles to the pathogenesis of the MS clinical spectrum in Austrian patients. Furthermore, we describe a patient with MS and pronounced psychiatric symptoms who is also a carrier of the pathogenic *C9orf72* HRE which we interpret most likely as coincidental. However, this case is exemplary of the increasing evidence and recognition of mixed neurodegenerative pathologies as patients get older these days [35,36]. The cases described in this study underline the need for further research on specific in vivo biomarkers and clinical red flags and, eventually, the importance to consider additional diagnoses in the context of atypical or rapid disease progression. Deciphering whether cognitive impairment is due to neurodegenerative changes in the course of MS itself or comorbid occurrence of neurodegenerative dementia will become increasingly important in order to provide optimal patient counseling and therapy.

## 4. Materials and Methods

### 4.1. Patient Cohort

DNA samples were derived from blood leukocytes of 382 unrelated patients diagnosed with MS from the biobank of the Department of Neurology of the Medical University of Vienna (MUV). A total of 643 DNA samples of neurologically unaffected individuals obtained from the biobank of the Department of Neurology of the MUV (n = 489) or acquired from the VITA collective (n = 154) served as healthy controls (HC). Written informed consent was obtained from all participants and ethical approval is available for all collectives. The study was approved by the ethics committee of the Medical University of Vienna (EK Nr 1220/2018). MS patients were clinically diagnosed based on established criteria [37] and assigned to sub-cohorts PPMS (n = 19), SPMS (n = 63) or RRMS (n = 269) according to their clinical phenotype [38]. A total of 31 patients could not be assigned to a distinct phenotype due to loss of follow-up. Information on patient characteristics is shown in Table 3.

### 4.2. C9orf72 Genotyping

DNA was isolated from peripheral whole blood using standard protocols. *C9orf72* genotyping was performed as described before [14]. In short, a two-step PCR assay was used to amplify the repeat sequence: In the first step, the complete 5′UTR of the *C9orf72* gene was amplified using primers flanking the pathological repeat region, followed by repeat primed PCR (RP-PCR). To validate *C9orf72* repeat expansions in individuals showing a characteristic chainsaw pattern in the RP-PCR analysis and to estimate the repeat length, Southern blotting was performed (see Supplementary Figure S1).

### 4.3. Statistics

For non-pathogenic range (2–30 repeats), we performed a Mann–Whitney U-Test to evaluate repeat length distribution between cohorts. Fisher’s exact test was used to compare frequencies of intermediate repeat alleles at different cut-offs (≥7, ≥17, ≥20, and ≥24). Correction for multiple testing was performed using Dunn’s multiple comparison test and Holm–Sidak correction for contingency analysis. Statistical analyses were performed using the software GraphPad Prism v8.0.0. A *p*-value of <0.05 was considered statistically significant.

## Figures and Tables

**Table 1:** Frequency of *C9orf72* repeat lengths in MS sub-cohorts.

	HC	PPMS	SPMS	RRMS	Not Assigned
Sample size	643	19	63	269	31
Mean repeat size (range)	4.4 (2–27)	4.0 (2–10)	4.8 (2–22)	4.3 (2–18)	3.9 (2–11)
Adjusted *p* value	-	0.90	0.77	0.83	0.61
S Allele (%)	1277 (99.3)	38 (100)	123 (97.6)	538 (100)	62 (100)
I Allele (%)	9 (0.7)	-	2 (1.6)	-	-
E Allele (%)	-	-	1 (0.8)	-	-

Abbr.: HC = healthy controls; PPMS = primary progressive multiple sclerosis; SPMS = secondary progressive multiple sclerosis; RRMS = relapsing remitting multiple sclerosis; S = small allele (<20); I = intermediate allele (20–30 repeats); E = expanded allele (>30 repeats). Differences in repeat length were analyzed by comparing HC to the distinct MS subtypes using the pairwise Mann–Whitney U test followed by Dunn’s correction for multiple testing. A total of 31 patients could not be assigned to a distinct phenotype due to loss of follow-up.

**Table 2:** Intermediate repeat lengths.

	HC	MS	*p* Value
Allele frequency ≥ 7			
S Allele (*n*, %)	1017 (79.1)	598 (78.4)	0.74
I Allele (*n*, %)	269 (20.9)	165 (21.6)	
Allele frequency ≥ 17			
S Allele (*n*, %)	1272 (98.9)	756 (99.2)	0.64
I Allele (n, %)	14 (1.1)	6 (0.8)	
Allele frequency ≥ 20			
S Allele (n, %)	1277 (99.3)	761 (99.7)	0.23
I Allele (n, %)	9 (0.7)	2 (0.3)	
Allele frequency ≥ 24			
S Allele (n, %)	1283 (99.8)	762 (100)	0.30
I Allele (n, %)	3 (0.2)	0	

Abbr.: HC = healthy controls; MS = multiple sclerosis; S = small allele; I = intermediate allele.

**Table 3:** Characteristics of study participants.

	HC	PPMS	SPMS	RRMS	Not Assigned
Sample size	643	19	63	269	31
Females	52%	58%	65%	70%	65%
Mean age at diagnosis (IQR) *	60.3 (43–76)	46.6 (41–52)	45.8 (38–53)	36.1 (28–44)	37.3 (24–48)
Mean EDSS (IQR) **	n.a.	4.4 (3.3–6.1)	5.5 (4.0–7.0)	2.1 (1.0–3.0)	0.8 (0–1.8)

* Mean age at diagnosis in HC refers to mean age at blood draw; ** EDSS was available for n (PPMS) = 10, n (SPMS) = 46, n (RRMS) = 177 patients; Abbr.: HC = healthy controls; PPMS = primary progressive multiple sclerosis; SPMS = secondary progressive multiple sclerosis; RRMS = relapsing remitting multiple sclerosis; EDSS= expanded disability status scale score; IQR = interquartile range.

## Data Availability

The data that support the findings of this study are available on request from the corresponding author, E.S.

## Supplementary Materials

The following supporting information can be downloaded at: https://www.mdpi.com/article/10.3390/ijms241411254/s1.
