# Supplementary material for: No Association of Multiple Sclerosis with C9orf72 Hexanucleotide Repeat Size in an Austrian Cohort
Source: Int J Mol Sci. 2023 Jul 9;24(14):11254. doi: 10.3390/ijms241411254 (PMC10378763; doi:10.3390/ijms241411254)
Supplement: Supplementary file 1 [file ijms-24-11254-s001.zip › ijms-2470877-supplementary.pdf]

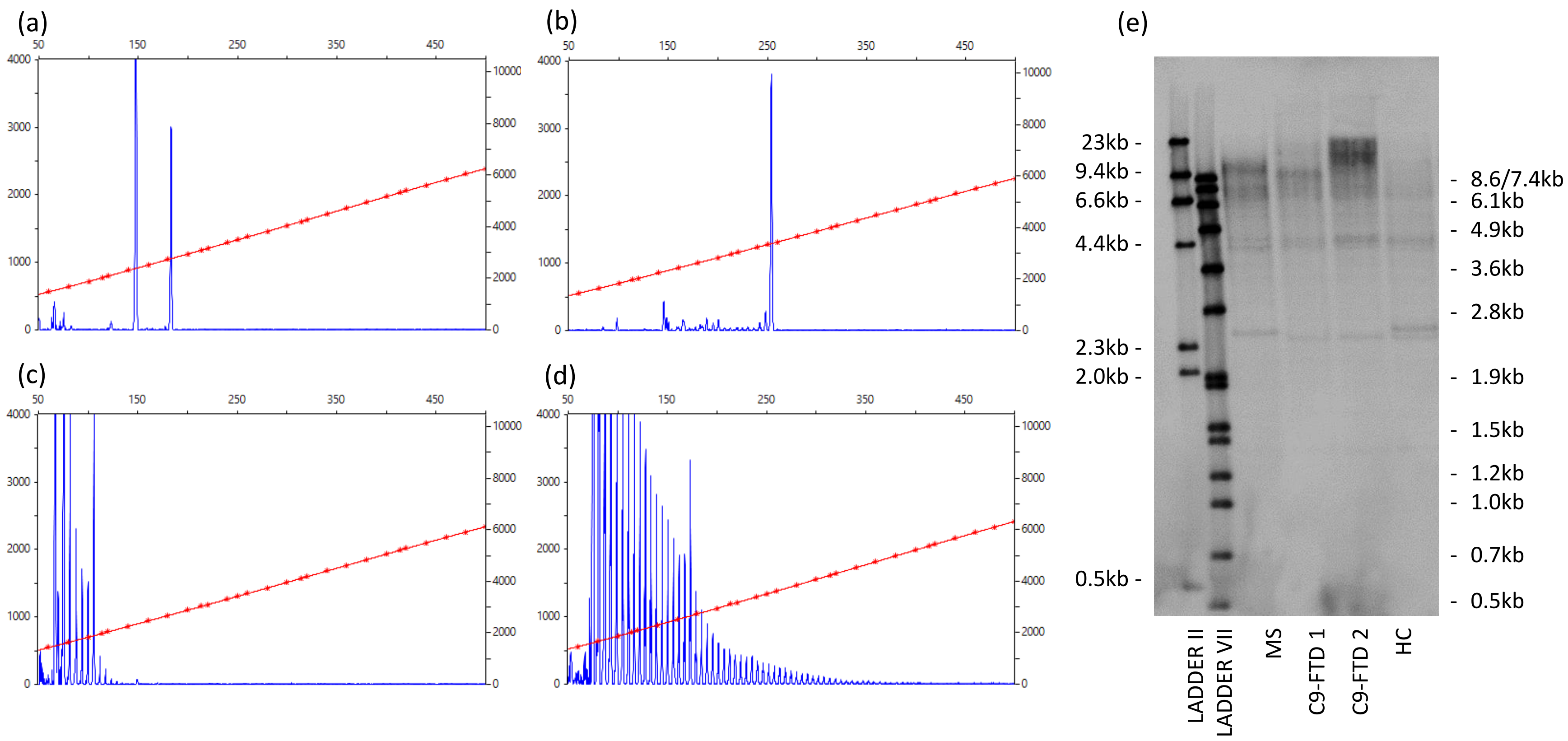

Supplementary Figure S1. Detection and sizing of the *C9orf72* repeat expansion

Genotyping was performed using a 2-step PCR protocol, as shown above on the example of a healthy control (panel a and c) and the MS patient carrying the repeat expansion (panel b and d). The electropherogram of the flanking PCR showed (a) two peaks (2 and 8 repeats) within the non-expanded range in the healthy control and (b) only one peak corresponding to an intermediate size of 20 repeat units in the *C9orf72* repeat expansion carrier. The following repeat primed PCR detected no repeat expansion in the (c) healthy control and (d) a characteristic chainsaw pattern in the mutation carrier. (e) Results were confirmed by Southern blotting of 2.5µg genomic DNA. The *C9orf72* repeat expansion carrier identified in the MS cohort showed a band above 10kb, corresponding to about 1300 repeat units. Two positive controls (C9-FTD 1 and C9-FTD 2) showed bands corresponding to 1100 and >3000 repeats, respectively. All individuals showed one (expansion carriers) or two (healthy control) discrete bands in the non-expanded range concordant to the size detected in the flanking PCR (above 2.3kb). Unspecific bands were observed at 4kb and 7kb. DIG-labelled DNA Molecular Weight Marker II and VII (Roche) were used to size the approximate repeat length. y axis = relative fluorescence units; X axis = base pairs.
